# Clinical and epidemiologic characteristics of non Hodgkin’s lymphoma in Bedouins in the south of Israel

**DOI:** 10.1186/2193-1801-2-672

**Published:** 2013-12-16

**Authors:** Itai Levi, Michal Feuchtwanger, Anat Rabinovich, Itamar Grotto

**Affiliations:** Hematology Institute, Soroka University Medical Center, Beer-Sheva, Israel; Department of Family Medicine, Faculty of Health Sciences, Ben-Gurion University of the Negev, Beer-Sheva, Israel; Department of Epidemiology, Faculty of Health Sciences, Ben-Gurion University of the Negev, Beer-Sheva, Israel; Department of Hematology, Soroka University Medical Center, PO Box 151, Beer-Sheva, Israel

**Keywords:** Non Hodgkin’s lymphoma, Bedouins, Epidemiology, Prognosis

## Abstract

The presentation of Non Hodgkin’s Lymphoma (NHL) may vary in relation to ethnicity or environmental effects. The objective of our study was to characterize NHL among the Negev Bedouin population, compared to the Jewish population.

Retrospective data were collected for 45 Bedouin and 45 Jewish patients who were treated at Soroka University Medical Center between 1990 and 2007.

Upon diagnosis, rates of aggressive NHL types, involvement of extra-lymphatic organs, as well as mean LDH were higher among Bedouin. Treatment response was poorer among the Bedouin: overall survival and disease-free survival were higher among Jews (mean of 145 and 168 months, respectively, compared with 70 and 45 months, respectively, among Bedouin; *p* < 0.01 for both).

In summary, disease aggressiveness and poor response to treatment among the Negev Bedouin may shed some light on the pathogenicity of NHL and may indicate a need for more aggressive treatment in different ethnic groups.

**Electronic supplementary material:**

The online version of this article (doi:10.1186/2193-1801-2-672) contains supplementary material, which is available to authorized users.

## Background

Non Hodgkin’s Lymphoma (NHL) is a heterogeneous group of lymphoid malignancies that differ in cell source and histology, as well as clinical characteristics, prognosis, and response to treatment. A worldwide significant increase in the incidence of the disease has been observed in the last forty years (Greenlee et al. 2007; ^a^). The Bedouin population in the Negev shows characteristics of Hodgkin Lymphoma that are different than those in the Jewish population (Levy et al. 2002). However, there is no knowledge regarding NHL characteristics at presentation, its response to treatment, and course in Bedouins in the Negev.

The Bedouin population in the Negev lives in permanent towns and small unrecognized villages. Due to the natural loss of livelihood, based in the past on agriculture and pasturing livestock, along with the lack of infrastructure and industry in the Bedouin towns, the Bedouin population is in the lowest level of the socioeconomic scale in Israel ^b^.

Cure of NHL is largely affected by characteristics of the disease at diagnosis, which can be expressed using the International Prognostic Index (IPI) (The International Non-Hodgkin’s Lymphoma Prognostic Factors Project 1993). Another important factor is the ability to tolerate aggressive treatment, which requires a high level of compliance and appropriate living conditions and sanitation, which are unavailable in many Bedouin villages.

A review of the literature suggests that although some characteristics of NHL were studied among Arab populations (Ameen et al. 2010; Castella et al. 2001; Cohen et al. 1989), clinical and epidemiological characteristics and the response to treatment of NHL among the Bedouin population have not been investigated thoroughly. A study that examined the clinical and epidemiological characteristics of Jewish and Bedouin patients with Hodgkin’s Lymphoma in southern Israel showed that Bedouin response to treatment is poorer compared to that of Jews (Levy et al. 2002). Another study showed a link between residential proximity to the Ramat Hovav industrial zone and increased risk of NHL (Dreiher et al. 2005). Ramat Hovav is a central industrial zone with 17 chemical factories and a toxic waste treatment site that may contaminate the environment with chemicals such as phenoxyacetic acid, organic solvents, triazines, and carbamates. While there is only a small Jewish population residing close to Ramat Hovav, a significant part of the Bedouin population resides close to it, and it is important to study the incidence of NHL among them.

Soroka University Medical Center is a tertiary center providing medical care to a major part of the Bedouin population in Israel. The purpose of this study was to characterize NHL in terms of the aggressiveness of the disease and its response to treatment in the Bedouin population compared to the Jewish population in the south of Israel.

## Results

Both study groups included 45 patients, of whom there were 21 men and 24 women. The mean age at diagnosis was 48 years (Table 1). A comparison of demographic and socioeconomic status between the groups showed Bedouin patients had larger families, with a mean of seven children compared to two among Jewish families. All 45 Jewish patients reside in houses. In contrast, among the Bedouin only 25 (55.6%) of the patients reside in houses, and 7 (15.5%) of them live in a hut or tent. In 13 Bedouin patients it was impossible to locate a residential address, which suggests that they lived in unrecognized villages. It was impossible to evaluate from the medical records the degree of chemical exposure and the dietary habits in both groups.

**Table 1:** **Comparison of demographic features between Bedouin and Jewish patients**

Demographic		Jews		Bedouins		***p*** value
		no.	%	no.	%	
Gender						
	Female	24	53.33	24	53.33	
	Male	21	46.67	21	46.67	
Mean age at diagnosis (years)		48.7		47.6		0.843
Mean number of children		2.4		7.1		<0.001
Housing form						
	House	45	100	25	55.6	
	Shed	0		6	13.3	
	Tent	0		1	2.2	
	Unknown	0		13	28.9	

The characteristics of NHL at diagnosis are worse among Bedouins than Jews (Tables 2 and 3). The incidence of aggressive NHL types is higher among Bedouin: 22% of the Bedouin compared to 9% of the Jewish patients (*p* = 0.03). Indolent lymphoma was found in 31.1% of the Jewish group, whereas it was found in only 11% of the Bedouin group. Rate of extra-nodal involvement is also higher among Bedouins (15%) compared with Jews (2%). The mean Lactate Dehydrogenase (LDH) level at diagnosis is higher among Bedouins (773 U/L) compared to a lower level (512 U/L) among Jews (*p* = 0.008). When comparing functional status according to the ECOG performance status between groups, the rate of patients in ECOG 3–4 at diagnosis is higher among Bedouin (24.4%) than Jewish patients (4.8%), *p* = 0.01. In addition, a larger number of Bedouins were diagnosed at stage 4 (40%) compared with Jews (24.4%), but the difference is not statistically significant.

**Table 2:** **Comparison of histological subtypes at diagnosis between Bedouins and Jews**

	Bedouins		Jews	
	No.	%	No.	%
Follicular	2	4.4	12	26.7
Marginal zone	3	6.6	2	4.4
Diffuse large B-cell	28	62.2	27	60
Mantle cell	2	4.4	1	2.2
Peripheral T-cell	5	11.1	-	-
Burkitt	2	4.4	1	2.2
T-cell lymphoblastic	3	6.7	2	4.4

**Table 3:** **Comparison of disease characteristics at diagnosis between Bedouins and Jews**

		Bedouins		Jews		***p*** value
		No.	%	No.	%	
All histological subtypes						
Stage 4		18	40	11	24.4	0.347
Extranodal involvement		7	15.6	1	2.2	0.026
	Stomach	2				
	Pleura	2				
	Liver	2				
	Lung	1				
	CNS			1		
Mean LDH (U/L)		773.92		512.15		0.008
ECOG 1–2		34	75.6	40	95.2	
ECOG 3–4		11	24.4	2	4.8	0.01
		Positive/tested				
Hepatitis B		3/11		0/11		
Hepatitis C		0/7		0/11		0.214
HIV		0/4		0/4		
DLBCL subtype						
Mean LDH (U/L)		827.07		538.17		0.019
Mean IPI		2.26		1.41		0.011
ECOG 1–2		22	73.3	25	96.2	0.022
ECOG 3–4		8	26.7	1	3.8	

Patients from both groups were treated similarly. The patients with Diffuse large B cell lymphoma (DLBCL) were treated with chemotherapeutic drugs alone–CHOP (Cyclophosphamide Hydrochloride Doxorubicin, Oncovin, prednisone) protocol until 2000 and R (Rituximab)-CHOP since 2000. The other patients were treated according to the acceptable chemotherapy protocol at the time of diagnosis with no difference between Jewish and Bedouin patients. The follow-up period for OS and DFS evaluation was the same for the two groups.

Comparing the response to treatment (Table 4), Jewish patients achieved a higher rate of CR (80%) compared with Bedouin patients (65.8%). In addition, the rate of relapse among patients who achieved remission is higher among Bedouins than Jews, 34.3% and 18.9%, respectively. This difference is not statistically significant.

**Table 4:** **Comparison of treatment outcome between Bedouins and Jews**

		Bedouins		Jews		***p*** value
		No.	%	No.	%	
Remission						
	Yes	25	65.8	32	80	
	No	10	26.3	5	12.5	
	Partial	3	7.9	3	7.5	0.290
Relapse						
	Yes	12	34.3	7	18.9	
	No	23	65.7	30	81.1	0.139
Mean overall survival (months)		70 (95% CI: 43–96)		168 (95% CI: 144–192)		<0.001
Mean disease-free survival (months)		57 (95% CI: 32–83)		145 (95% CI: 116–175)		<0.001
DLBCL patients						
Mean overall survival (months)		88 (95% CI:54–122)		153 (95% CI:118–188)		0.054
Mean disease-free survival (months)		75 (95% CI:41–108)		136 (95% CI:98–174)		0.046

The mean OS for all patients in our series was 122 months (95% CI: 99–145 months). More than half of all patients were alive at the end of the follow-up period. The mean DFS for all patients was 105 months (95% CI: 83–129 months) and median DFS was 76 months (95% CI: 39–113 months).

**Figure 1:**
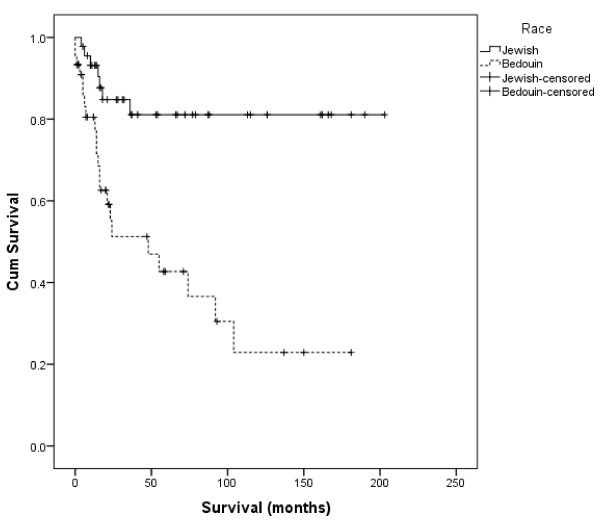
**Overall survival in months by ethnicity (Jewish versus Bedouin).**

Comparison of OS between the two study groups (Figure 1 and Table 4) shows OS to be lower among Bedouin. Mean OS among Jews was 168 months (95% CI: 144–192 months), compared to 70 months in Bedouin (95% CI: 43–96 months). The difference is statistically significant (*p* < 0.001).

**Figure 2:**
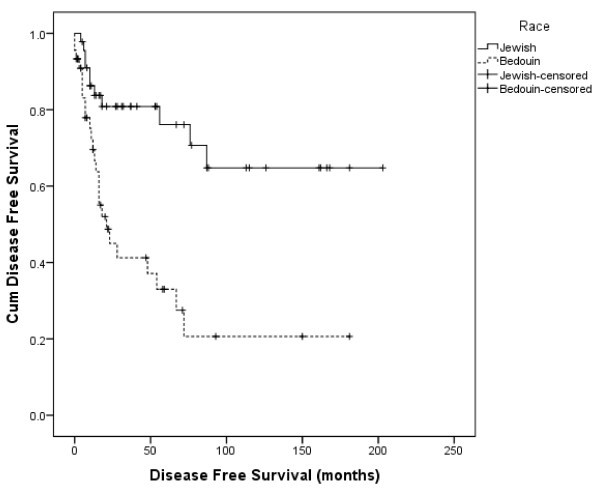
**Disease-free survival in months by ethnicity (Jewish versus Bedouin).**

When comparing DFS between the study groups (Figure 2 and Table 4), it was found to be significantly higher in Jews, with a mean of 145 months (95% CI: 116–175 months) compared to 57 months (95% CI: 32–83 months) in Bedouins.

**Figure 3:**
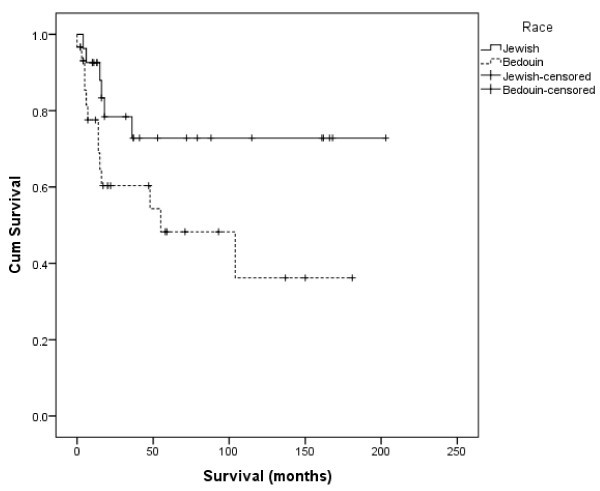
**Overall survival by ethnicity (Jewish versus Bedouin), only among patients with Diffuse Large B-Cell Lymphoma.**

**Figure 4:**
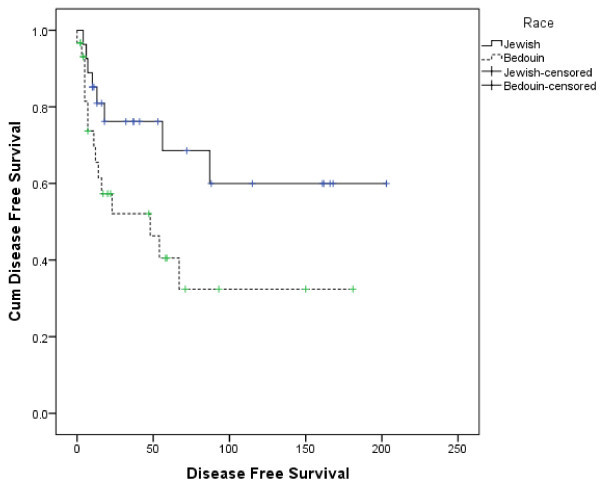
**Disease-free survival in months by ethnicity (Jewish versus Bedouin), only among patients with Diffuse Large B-Cell Lymphoma.**

Since we found a different distribution of pathologic diagnoses between Jews and Bedouins, we assumed that this had an effect on other characteristics of the disease at diagnosis (such as performance status, stage of disease, IPI score). To test whether differences in the characteristics of Lymphoma exist between study groups regardless of pathology type, we compared a subgroup of patients with the same pathology. We compared patients diagnosed with DLBCL, who comprise 60% of patients in this series (Table 2). The differences in expression of the disease (IPI, LDH, performance status) were persistent between the two sub-groups, being significantly worse among the Bedouin. Comparison of OS and DFS between Jews and Bedouin with DLBCL yielded similar results to those for all patients. OS and DFS were lower among the Bedouin, with borderline significance (Table 4, Figures 3 and 4).

## Discussion

In this study we found that NHL has different characteristics among the Bedouin compared to Jewish population in the south of Israel. Disease characteristics at diagnosis tend to be worse among the Bedouin. Extra-nodal involvement, high LDH, and poor performance status are more frequent among the Bedouin. Advanced disease at presentation is more common among the Bedouin, but the difference is not significant. The incidence of aggressive lymphomas is also significantly higher among Bedouin. Jews have a higher rate of follicular lymphoma whereas the Bedouins have higher rates of T-cell Lymphoma and other aggressive pathologies.

Similar findings were found in a study conducted at Ben-Gurion University (Levy et al. 2002). The study compared characteristics of Hodgkin’s Lymphoma between Bedouin and other populations in southern Israel. The characteristics of the disease at diagnosis were different between groups. Histology of mixed cellularity and more advanced disease at diagnosis were more common among the Bedouin. Results for treatment response and prognosis were also similar to those seen in the current study. Bedouin had a higher rate of resistant disease, and lower overall survival and survival without disease.

There are several possible explanations for these differences in the presentation of Lymphoma among Bedouin in this study.

Lower availability and reduced use of medical services among the Bedouin result in the diagnosis of Lymphoma at a later stage. This is a result of low socioeconomic level, low awareness of preventive medicine, and lack of access to medical service.

It also may be possible that exposure to chemical contaminants plays a role in the development of Lymphoma. As noted above, there is a study that examined the relationship between residential proximity to Ramat Hovav and incidence of NHL (Dreiher et al. 2005). The study found that living near the Ramat Hovav site constitutes a risk factor for morbidity in NHL. The study was conducted among a population that included mostly an urban Jewish population. Presumably Bedouin, who live even closer to the site, are more exposed to these toxic substances. However, it is impossible to estimate with certainty how many Bedouin who were included in our study have been exposed to environmental pollutants near their residence.

An important factor in development of some malignant disease is nutrition habits. Two studies (Fraser et al. 2001, 2008) compared diet and eating patterns among Jewish and Bedouin populations in southern Israel. Bedouin nutrition was found to be characterized with lower intake of fat, cholesterol, and protein and higher intake of carbohydrates and fibers. However, there are no public data regarding the influence of this diet on NHL severity or survival.

It might be that genetic and geographic differences play a role in the differences between the groups, mainly in the histological type of the Lymphoma. T-cell Lymphomas, for instance, are more common in Asia, while the B-cell Lymphomas are more common in Western countries (Rüdiger et al. 2002).

A study from the north of Israel showed higher prevalence of nodular lymphoma in Ashkenazi Jews and higher prevalence of extranodal lymphoma in Arabs and non-Ashkenazi Jews (Cohen et al. 1989). A study that evaluated frequencies of NHL subtype in Kuwait found a higher prevalence of DLBCL and extranodal presentation compared to the Western world (Ameen et al. 2010). A study from Jordan classified 111 cases of NHL from two major medical centers (Almasri et al. 2004). Aggressive lymphoma accounted for the majority of NHL, while indolent lymphomas were rare and accounted for less than 15% of all NHLs. Our results are similar to these results. Higher incidence of aggressive lymphomas such as Burkitt’s and Lymphoblastic lymphoma in the Bedouin as in other Arab populations may have a common mechanism. This may indicate genetic and environmental factors that are responsible for the greater presentation of aggressive histological subtypes in Bedouin compared to the Jewish population. Because of the small number of patients in this study, we cannot evaluate accurately how this study represents the distribution of histological subtypes of Lymphoma among all the population, and especially among the Jewish population in Israel.

Our results show decreased response to treatment among the Bedouins. OS and DFS are significantly better among Jews compared to Bedouins. These results can be explained by two main reasons: more aggressive and advanced disease at diagnosis and lower compliance with treatment compared to Jewish patients, partly because of poor living conditions. Two studies from the south of Israel (Tamir et al. 2007; Cohen et al. 2008) found that low compliance with drug therapy and follow-up tests, and non-attendance to the clinic are major problems in the Bedouin population. These findings may suggest that an important reason for lower response rate of Bedouin to chemotherapy is poor compliance.

Due to the different distribution of histological subtypes between the groups, and the influence of histology on prognosis, we decided to test whether differences between populations are maintained when comparing subgroups of patients with identical pathology. The characteristics of DLBCL at presentation are significantly worse among Bedouins (mean LDH was higher, poorer performance status, and higher IPI). Similarly, the survival was lower among the Bedouins, with borderline significance.

There are several limitations to this study. There was no randomization in selecting populations. The study group included all the Bedouin patients who were found suitable for inclusion. The comparison group was selected randomly by matching age, sex, and year of diagnosis to those of the study group. This method may have created selection bias. We do not know whether the comparison group represents the entire Jewish population of NHL patients in the Negev. We might have found a higher rate of aggressive lymphoma if we reviewed all records of Jewish NHL patients. In addition, we do not know whether there is a difference in the age of lymphoma onset between the two populations.

As is known, the characteristics of NHL at diagnosis are predictors of the response to treatment. In the current study we found that NHL indices among the Bedouin at diagnosis were worse, in accordance with lower response rate to treatment and lower survival. Because we did not compare response to treatment in patients with the same IPI, we cannot determine whether the lower survival rate among the Bedouin is due to different characteristics of the lymphoma at diagnosis or whether the difference may also depend on other factors affecting the course of the disease. This comparison could not be done due to the small size of the sample.

In summary, NHL has different characteristics among the Bedouins in the Negev compared to Jews. Among the Bedouins, the disease tends to emerge in later stages, with a higher IPI and more aggressive appearance. The response to treatment and survival rate is lower among the Bedouins.

The inverse relationship between socioeconomic level and morbidity and mortality is known for many diseases. Our study results are compatible with this finding. Authorities should invest in establishing a medical infrastructure available to this population, together with an educational program to raise awareness for early detection and treatment of diseases. Medical staff should be aware of the difficulties for parts of the Bedouin population in approaching medical services. This requires attention and effort, but will certainly increase the number of patients diagnosed at the initial stages of disease. As the availability of medical care and compliance of this population to treatment will increase as a result of education, we hope that the outcome of treatment will also improve.

## Methods

A retrospective study was conducted. Data for all Bedouin patients diagnosed with NHL and treated at Soroka University Medical Center between 1990 and 2007 was collected and compared to data of a group of Jewish patients from the same region treated in our center. The study was approved by the Institutional Review Board of Soroka University Medical Center.

Patients (Bedouin and Jewish) were identified through the Israel Ministry of Health Cancer Registry. The study group included all Bedouin patients with a histological diagnosis of NHL, over 18 years-of-age at diagnosis, treated and followed at Soroka University Medical Center, and with accessible medical records. Forty-five Bedouin patients met the inclusion criteria. For each Bedouin patient, a Jewish patient was matched for sex, age (±2 years), and year of diagnosis (±2 years). In cases where there was more than one possible control, the final control was selected randomly. For each patient we retrieved clinical, laboratory, and imaging data at the time of diagnosis, and the response to treatment. The two groups were compared according to clinical and laboratory characteristics, aggressiveness, and response to treatment. Aggressiveness was categorized according to the histological sub-type of NHL. Burkitt’s lymphoma, Lymphoblastic lymphoma, T cell lymphoma, and Mantle cell lymphoma were defined as aggressive lymphomas. DLBCL was defined as intermediate grade lymphoma, and marginal zone lymphoma and follicular lymphoma were defined as low grade lymphomas.

Response to treatment was defined by the rate of complete remission (CR), partial remission (PR), disease-free survival (DFS), and overall survival (OS). Treatment response was assessed according to the International Working Group criteria published in 1999 (Ceason et al. 1999). A CR was defined as the disappearance of all previously measurable lesions and the absence of any new tumor lesions. PR was defined as a decrease of at least 50% of each lesion. Progressive disease or relapse was defined as more than 50% increase of at least one lesion, or the presence of newly developed lesions.

### Statistical analysis

For comparison of the aggressiveness variables between the study groups, we calculated statistical significance using chi-square test for categorical variables and *t*-test for continuous variables. We calculated overall and disease-free survival by the Kaplan-Meier method and compared Bedouin and Jewish patients using Log-rank test. All analyses were done with SPSS software, version 15.0.

## Endnotes

^a^Israel Cancer Registry, Trends of Non Hodgkin’s Lymphoma 1980–2007. August 2010. [http://www.old.health.gov.il/download/sartan/trends/NHL.pdf].

^b^Israel Central Bureau of Statistics: Local councils and municipalities by socio-economic index, ranking and cluster membership, 2003. In: Socioeconomic characteristics of Israeli local authorities, table two. October 2006. [http://www.cbs.gov.il/publications/local_authorities2003/pdf/t02.pdf].

## Authors’ original submitted files for images

Below are the links to the authors’ original submitted files for images. Authors’ original file for figure 1 Authors’ original file for figure 2 Authors’ original file for figure 3 Authors’ original file for figure 4
