# Supplementary figures and images for: Clinical and epidemiologic characteristics of non Hodgkin’s lymphoma in Bedouins in the south of Israel
Source: Springerplus. 2013 Dec 16;2(1):672. doi: 10.1186/2193-1801-2-672 (PMC3874137; doi:10.1186/2193-1801-2-672)

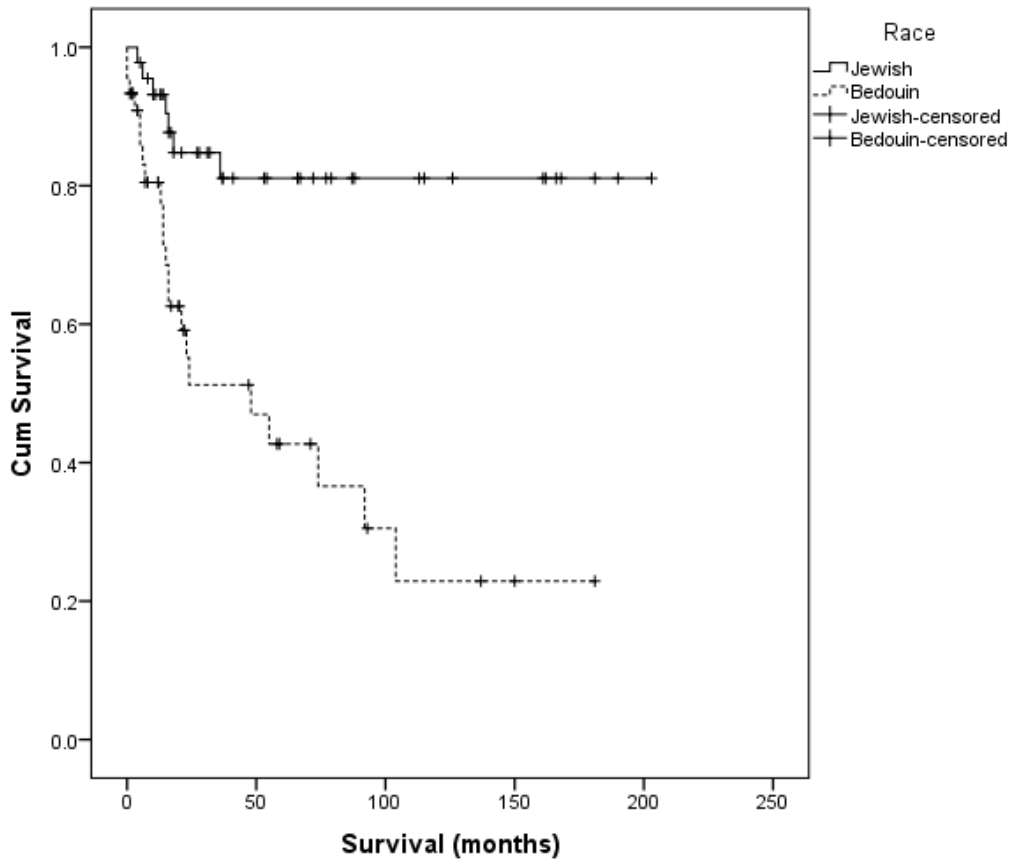

Supplement: Supplementary file 1 — Authors’ original file for figure 1 [file 40064_2013_744_MOESM1_ESM.pdf]

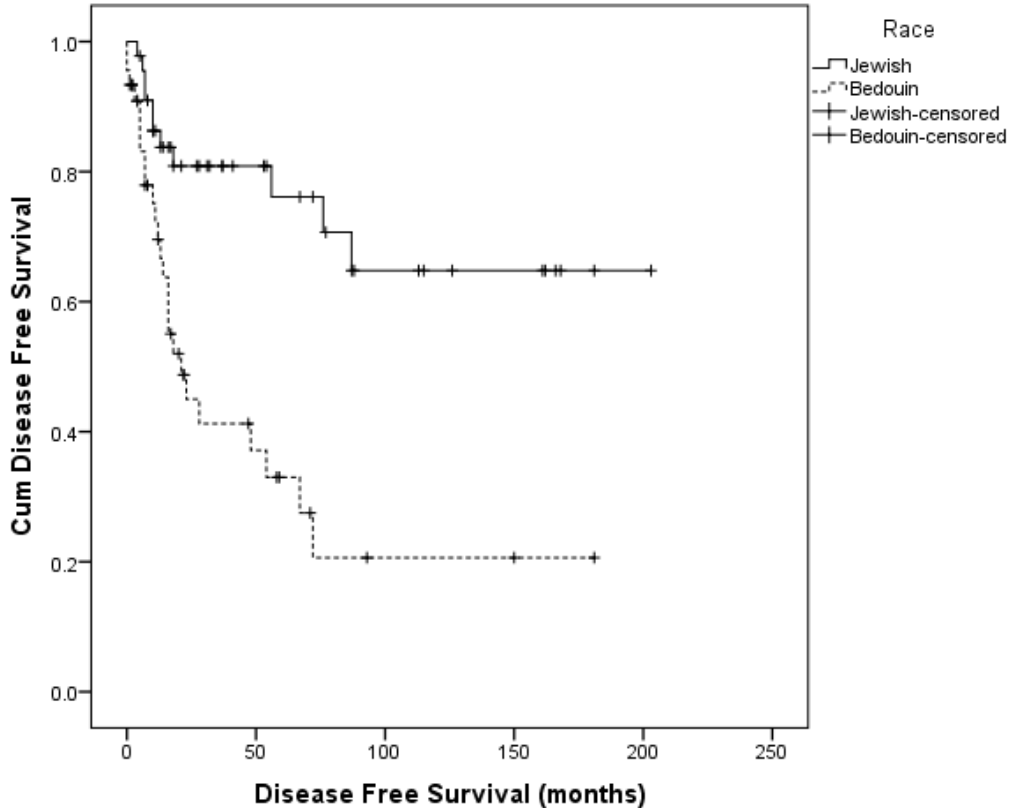

Supplement: Supplementary file 2 — Authors’ original file for figure 2 [file 40064_2013_744_MOESM2_ESM.pdf]

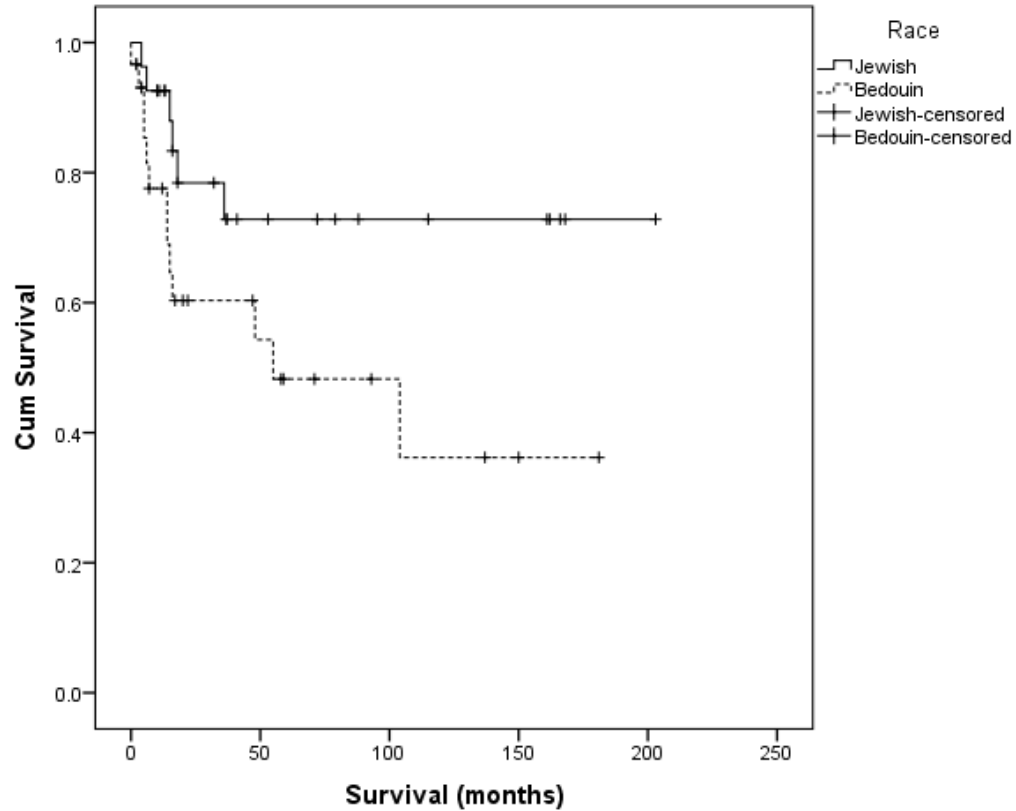

Supplement: Supplementary file 3 — Authors’ original file for figure 3 [file 40064_2013_744_MOESM3_ESM.pdf]

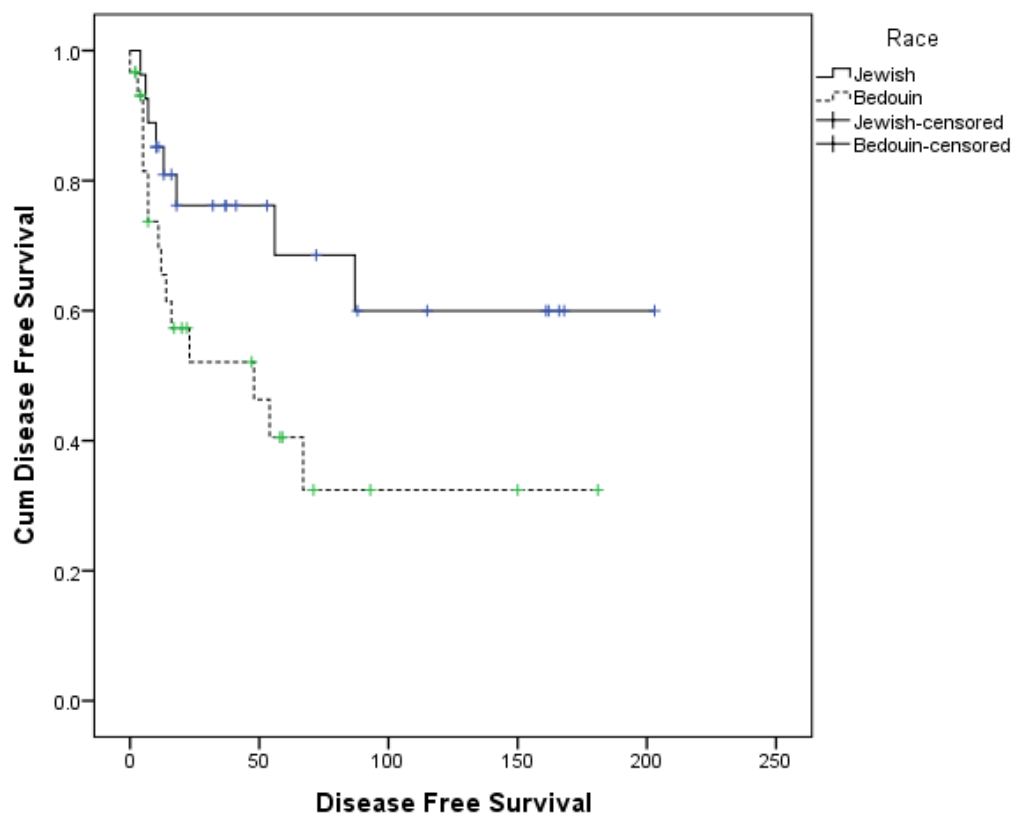

Supplement: Supplementary file 4 — Authors’ original file for figure 4 [file 40064_2013_744_MOESM4_ESM.pdf]
